# An Improved YOLOv7 Lightweight Detection Algorithm for Obscured Pedestrians

**DOI:** 10.3390/s23135912

**Published:** 2023-06-26

**Authors:** Chang Li, Yiding Wang, Xiaoming Liu

**Affiliations:** 1 College of Electrical and Control Engineering, North China University of Technology, Beijing 100144, China; lc0716@mail.ncut.edu.cn (C.L.);; 2 College of Information Science and Technology, North China University of Technology, Beijing 100144, China

**Keywords:** pedestrian detection, obscured pedestrian, mobilenetV3, attention mechanism

## Abstract

The detection algorithm commonly misses obscured pedestrians in traffic scenes with a high pedestrian density because mutual occlusion among pedestrians reduces the prediction box score of the concealed pedestrians. The paper uses the YOLOv7 algorithm as the baseline and makes the following three improvements by investigating the variables influencing the detection method’s performance: First, the backbone network of the YOLOv7 algorithm is replaced with the lightweight feature extraction network Mobilenetv3 since the pedestrian detection algorithm frequently needs to be deployed in driverless mobile, which requires a fast operating speed of the algorithm; second, a high-resolution feature pyramid structure is suggested for the issue of missed detection of hidden pedestrians, which upscales the feature maps generated from the feature pyramid to increase the resolution of the output feature maps and introduces shallow feature maps to strengthen the distinctions between adjacent sub-features to enhance the network’s ability to extract features for the visible area of hidden pedestrians and small-sized pedestrians in order to produce deeper features with greater differentiation for pedestrians; and the third is to suggest a detection head based on an attention mechanism that is employed to lower the confidence level of target neighboring sub-features, lower the quantity of redundant detection boxes, and lower the following NMS computation. The mAP of the suggested approach in this work achieves 89.75%, which is 9.5 percentage points better than the YOLOv7 detection algorithm, according to experiments on the CrowdHuman pedestrian-intensive dataset. The algorithm proposed in this paper can considerably increase the detection performance of the detection algorithm, particularly for obscured pedestrians and small-sized pedestrians in the dataset, according to the experimental effect plots.

## 1. Introduction

The application of pedestrian detection techniques in settings with dense pedestrian targets, such as retail malls, transit hubs, and unmanned vehicles, is a significant study area in the science of computer vision [1]. The high degree of pedestrian look resemblance, the wide range in size, and the frequent obstruction of pedestrians from one another in crowded scenes present difficulties for pedestrian recognition methods. The primary foundation for traditional pedestrian detection algorithms is manual feature construction [2,3], which has the drawbacks of slow speed and low accuracy. Deep learning-based pedestrian detection algorithms have been widely used in the fields of intelligent transportation, autonomous driving, and video detection with the advantages of simple model structure and high detection accuracy in recent years due to the huge popularity of convolutional neural networks in the field of computer vision.

The two primary categories of deep learning-based pedestrian detection algorithms are two-stage methods and one-stage methods. The R-CNN [4] series of algorithms, including R-CNN, Fast R-CNN [5], Faster R-CNN [6], etc., are typically used as representative algorithms. These algorithms have extensive computations, slow operation speeds, and great precision, making it challenging to meet real-time needs. The single-stage detection algorithm employs the concept of regression to input the image to be detected into the convolutional neural network without creating candidate regions, directly predicts the bounding box information of the target pedestrian in the image, discriminates the target class contained in the bounding box, and then regresses the position of the bounding box. The SSD [7] algorithm, the YOLO series [8,9,10,11,12,13,14] of algorithms, the RetinaNet [15] algorithm, and the CenterNet [16] algorithm are examples of this class of methods. The single-stage detection technique does not include the operation of generating candidate regions, which reduces the number of model parameters, speeds up model execution, and significantly advances pedestrian detection technology.

However, the current mainstream standard image processing algorithms and deep learning methods are still unable to adequately address three of the most challenging issues in pedestrian detection technologies. First, in dense pedestrian scenes, the limited visible pixel area of obscured and small-sized pedestrians can easily lead to missed and false detection of pedestrians. Second, the pedestrian features extracted by the detector will be more susceptible to interference from noise in the environmental background when there are pedestrians of various sizes in the scene or individuals of smaller size and lower resolution in the pedestrian target, easily leading to false detection of small-sized pedestrians. Third, the traditional non-maximum suppression (NMS) [17] method has the potential to improperly suppress substantially overlapping prediction frameworks, which is a surefire way to miss the detection of pedestrians who are obscured.

The literature [18] suggests the Soft NMS method, which appropriately reduces the target confidence level according to the degree of overlap and then completes the screening of redundant target frames by specifying a confidence threshold, in order to address the issue that traditional NMS methods can incorrectly suppress highly overlapping targets. The literature [19] suggests an adaptive thresholding NMS method called Adaptive NMS, which bases its adaptive adjustment of the NMS thresholds of various prediction frames on the target overlap. The previously mentioned method can somewhat enhance the model’s performance for detecting pedestrians in dense scenarios, but when many prediction frames belonging to the same target are not tightly consistent enough, the rate of false positive detection results rises dramatically. Additionally, a few additional researchers have focused on researching feature extraction techniques for obscured objects. To enhance the ability to extract distinguishable depth features for highly overlapping targets, a multi-scale attention mechanism is proposed in the literature [20]. Furthermore, in the literature [21], an attention mechanism is added to the feature extraction network, and a feature extraction enhancement algorithm is proposed. Although the aforementioned algorithms have increased the capacity to detect obscured pedestrians, they are still unable to handle situations when pedestrians are heavily obscured by one another in crowded scenarios.

In light of the challenging issues of obfuscated pedestrians and small-sized pedestrian targets detection in dense scenarios, this paper proposes an enhanced YOLOV7 [14] pedestrian detection method for dense situations. First, we designed and constructed a streamlined feature extraction backbone network, replacing the heavy-duty network YOLOV7 with the light-weight network MobileNetV3 [22], which significantly reduces the number of model parameters and improves the model running speed. Then, in the feature fusion section, the feature maps are up-sampled to create a high-resolution feature pyramid network, and the distinguishability of adjacent sub-features is improved by fusing the detail information from the bottom layer to improve the network’s feature extraction capability for small-sized pedestrians and obscured pedestrians in shallow feature maps. In the head of the detection network, the enhanced dual efficient attention mechanism module ECSA (Efficient Channel and Spatial Attention Mechanism, ECSA) is integrated, which is used to lower the confidence level of the target’s surrounding sub-features and lower the quantity of redundant prediction frames. As a result, the main contribution of this paper is that it increases the resolution of the feature pyramid feature map and embeds an improved attention mechanism module in the detection head. The detection accuracy of the model for obscured pedestrians is improved, while the number of parameters required by the model is reduced. We conducted experiments on CrowdHuman [23], the most convincing dense obstruction target dataset, to confirm the efficacy of the aforementioned improvements. The results of the experiments demonstrate that the proposed algorithm can be used to successfully enhance YOLOv7’s detection ability for obscured pedestrians and small pedestrians.

## 2. Materials and Methods

### 2.1. Dataset

On the public datasets Wider-Person and CrowdHuman, we experimentally validated the usefulness of the proposed method used in this research. In this study, using the public datasets CrowdHuman and Wider-Person, we experimentally validated the effectiveness of the suggested algorithm. With a training set of 15,000 images, a test set of 5000 images, a validation set of 4370 images, and a total of 470,000 instances in the training and validation sets, CrowdHuman is a sizable public dataset for pedestrian detection. On average, there are about 23 pedestrians in each image. With an average of 2.4 boxes with IoU larger than 0.5 per image and a variety of obstructions between rows at the same time, the density of pedestrian occurrences is higher than in other datasets. Therefore, for the obstructed pedestrian detection issue, the experimental results based on this dataset are more compelling. The Wider-Person [24] dataset, which contains 13,382 photos and 400,000 human targets in all with varying degrees of obstruction, serves as a benchmark dataset for outdoor pedestrian detection. In addition, 9000 pedestrian photos with the appropriate labels are chosen for training and validation purposes in this research.

### 2.2. Structure of YOLOv7

**Figure 1:**
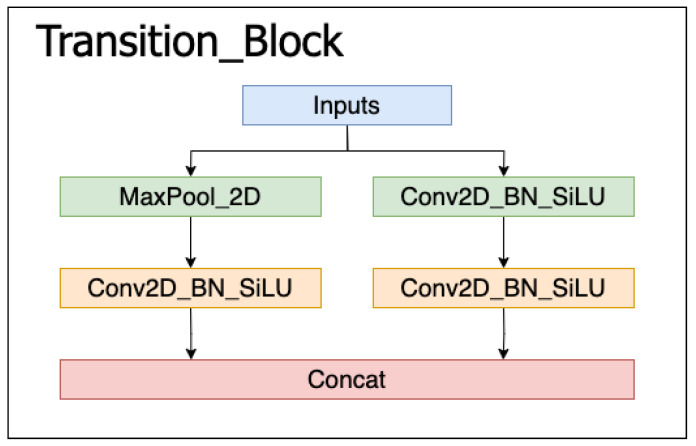
The MPConv module.

Following a number of updates to the YOLO series networks, the YOLOv7 algorithm structure is put forth. It not only keeps the real-time features of the YOLO series algorithms but also outperforms the majority of the current commonplace pedestrian detectors and has the highest average accuracy on significant image recognition datasets such as ImageNet [25] and MS COCO [26]. The detection concept of YOLOv7 is comparable to YOLOv4 [11], as are other network models from the YOLO series. Its model’s framework process is shown in Figure 1.

The input, backbone feature extraction network, feature pyramid network, and detection head are the four key components of the YOLOv7 detection network. After pre-processing such as cropping and data enhancement, the image to be detected is fed into the YOLOv7 backbone network, which performs initial feature extraction on this image to obtain a collection of three effective feature layers of different sizes: (80, 80, 512), (40, 40, 1024), and (20, 20, 1024). Following that, the shallow feature map of the image containing the shallow semantic information about the pedestrian will be fused with the three effective feature layers in the feature pyramid part to improve the performance of the network for pedestrian detection, and finally, the detection results will be output after the detection head.

**Figure 2:**
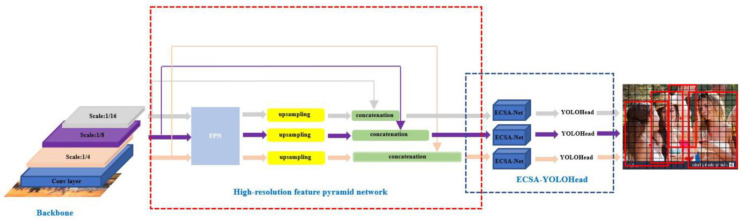
Diagram of the network architecture for our proposed algorithm, HRECSA-Net.

The general convolution module, E-ELAN (Extended-ELAN) module, MPConv module, and SPPCSPC module make up the majority of the YOLOv7 backbone feature extraction network. Based on the original ELAN, the e-ELAN module modifies the computational blocks while preserving the original ELAN’s transition layer structure and employing expand, shuffle, and merge cardinality to improve network learning without changing the gradient route. In order to overcome the issue of picture distortion brought on by down-sampling in the image feature extraction process and to address the issue of repeated features extracted from the image by the convolutional neural network, the SPPCSPC module adds concurrent multiple MaxPool operations to the convolution process. The MPConv module of the algorithm in YOLOv7 combines these two down-sampling strategies, as opposed to the typical down-sampling modules in backbone networks, which typically utilize a convolutional kernel of size 3 × 3 with a step size of 2 or a MaxPooling of size 2 × 2. According to Figure 2, the down-sampling module contains two branches: the left branch is a MaxPooling with a step size of 2 and a general convolution of 1 × 1, and the right branch is a general convolution of 1 × 1 with a convolution kernel size of 3 × 3 and a step size of 2. The output of the two branches is concatenated, which can increase the network’s generalizability. The MaxPooling operation broadens the perceptual field of the current feature layer and then fuses it with the feature data processed by the general convolution.

The YOLOv7 algorithm is identical to the YOLOv5 algorithm, which uses the traditional PANet [27] and FPN [28] structures, in the feature map fusion stage. Three different pedestrian-size detection heads—large, medium, and small—are used in the detection head section of YOLOv7, and the RepConv module’s structure differs slightly throughout network training and inference.

## 3. Improved YOLOv7

As shown in Figure 2, this paper optimizes YOLOv7 in three areas: replacing the backbone network, improving the feature pyramid structure, and designing the detection head based on the attention mechanism in response to the problems that the pedestrian tracking algorithm is prone to miss detection and false detection for obscured pedestrians and small-sized pedestrians in dense scenes, as well as the problem that the model is computationally expensive and requires high hardware equipment in dense scenes. Figure 2 displays the network’s architecture.

### 3.1. The Lightweight Backbone MobilemetV3

We use the lightweight backbone network MobilenetV3 to replace the original backbone network DarkNet53 of YOLOv7, which can not only reduce the computational cost of the detection model and the required storage space but also significantly improve the model’s accuracy for pedestrian detection. This reduces the number of parameters in the pedestrian detection model and the requirement of the algorithm for hardware devices.

**Figure 3:**
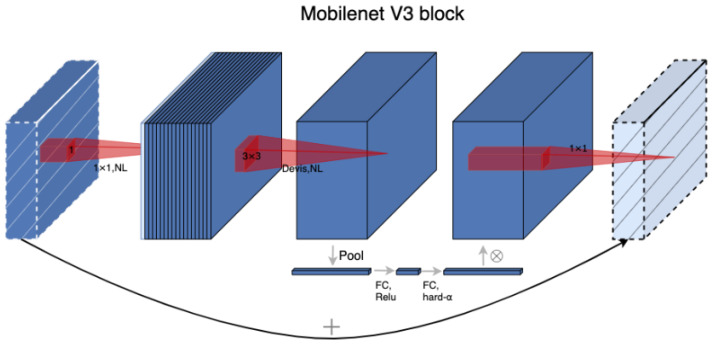
Diagram of the MobilenetV3 network architecture.

A lightweight deep neural network model called MobilnnetV3 was presented by Google in 2019. Its main concept is to use deep separable convolution to cut down on the number of network parameters. In addition to inheriting the inverse residual structure with linear bottleneck and deeply separable convolutional block from MobilenetV1 [29] and MobilenetV2 [30], MobilenetV3 also introduces the lightweight attention model (Squeeze-and-Excite, SENet) [31] structure and uses the h-swish activation function in place of the swish function to reduce the number of operations and enhance model performance. Figure 3 displays the network architecture of MobilenetV3. The MobilenetV3 network first utilizes 1 × 1 convolution to upscale the input image’s feature channels before extracting features using 3 × 3 depthwise separable convolution blocks. In the feature extraction process, the possibility of overfitting is diminished. where NL stands for nonlinear activation function, FC for fully connected operation, and Pool for downsampling. Dwise stands for depthwise separable convolutional block.

**Figure 4:**
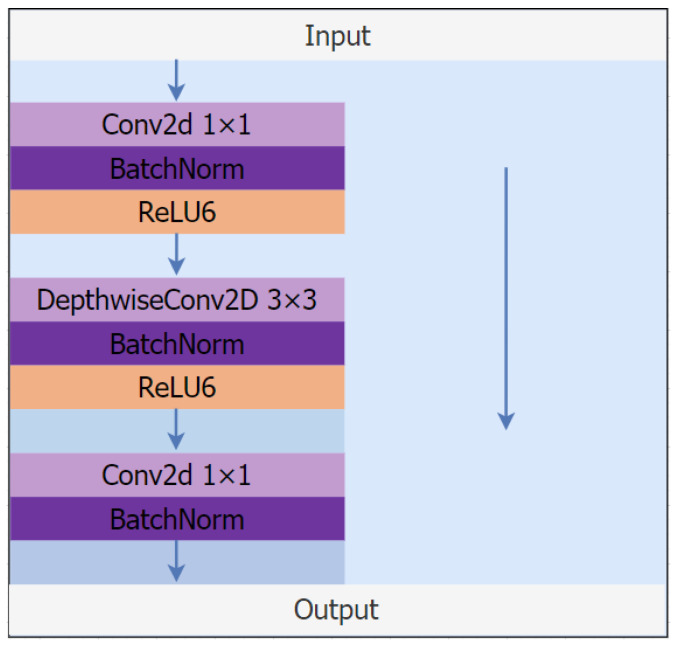
Diagram of a depthwise convolutional block structure.

The depthwise separable convolutional block network’s structure is depicted in Figure 4. The Mobilenet series network is mostly utilized to reduce the number of parameters needed by the depthwise separable convolutional blocks. Assuming a convolutional layer with 3 × 3 convolutional kernels, 16 input channels, and 32 output channels, the process of feature extraction would first involve traversing each of the 16 feature channels with 32 convolutional kernels of size 3 × 3, followed by the output of a feature map with 32 feature channels, and 16 × 32 × 3 × 3 = 4608. Following the deep separable convolution block, we first traverse 16 feature channels of the input feature map with 16 convolution kernels of size 3 × 3, resulting in 16 feature profiles, and then we traverse 32 feature channels with 32 convolution kernels of size 1 × 1, resulting in the output feature map with 32 feature channels, which is then fused with the residual edges. This procedure requires 16 × 3 × 3 + 16 × 32 × 1 × 1 = 656 parameters in total. By comparing the two sets of data, we can see that the deep separable convolutional block drastically lowers the number of model parameters while increasing the network’s processing performance.

### 3.2. High-Resolution Feature Pyramid Network

**Figure 5:**
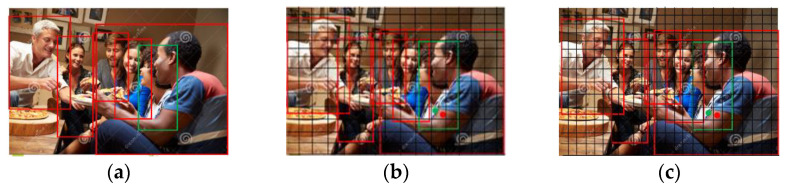
Difficulty in detecting pedestrians. (**a**) High overlap of six people; (**b**) the two strongly overlapping individuals on the right both have centroids that fit into the same grid; and (**c**) the centers of the two nearly identical individuals on the right fit into several grids.

Pedestrian detection techniques are typically used in regions with high traffic volumes, such as malls, transportation hubs, and junctions of roads. The foreground pedestrians occupy the majority of the visible pixels of the obscured pedestrians in the image because of the substantial mutual obstruction and the strong similarity of pedestrian appearance, as illustrated in Figure 5b. The foreground pedestrians are denoted by red boxes, whereas pedestrians who are obscured are denoted by green boxes, and their center points are denoted by red and green dots, respectively. The YOLOv7 detection network operates on the basis of a continuous resolution decrease of the input image after multiple iterations of downsampling by the backbone network, which causes the centroids falling into the same sub-feature region to be treated as identical pedestrians and sent to the training process, resulting in the missed detection of obscured pedestrians. Additionally, because the visible area of the obscured pedestrian is so small and the obscured pedestrian only occupies a small number of pixels, a significant amount of foreground pedestrian information is mixed in when the appearance features of the obscured pedestrian are extracted. This has an impact on the feature extraction of the obscured pedestrian and is a common reason why the obscured pedestrian is mistakenly detected.

This work suggests a high-resolution feature pyramid structure to overcome the aforementioned issues, and the output feature maps of the feature pyramid are upsampled. The shallow feature map and the up-sampled high-resolution feature map are then merged once more for feature fusion. Although the shallow feature map of pedestrians provides less in-depth semantic data, it allows the adjacent sub-features to incorporate more specific data with a higher degree of partitioning from the lower-level features. While increasing the variability between adjacent features in the feature pyramid, the higher-level semantic information of the deeper features is preserved. The centroids of the highly overlapping pedestrians fall as much as feasible into distinct sub-feature regions by raising the resolution of the feature maps. The high-resolution feature pyramid structure can improve the ability of the network to extract the features of the obscured pedestrians and reduce the likelihood of missed and false detection of the obscured pedestrians because the appearance features of the obscured pedestrians originate from their limited visible pixel areas, and the shallow feature maps have smaller sensory fields and contain more detailed information than the deep feature maps.

### 3.3. Improved Attention Mechanism

We presented a high-resolution feature pyramid structure above, where pedestrian features are divided into a denser grid by up-sampling to increase the resolution of the feature map generated from the feature pyramid network in order to decrease the probability of missing and false detection of hidden pedestrians. However, because the number of pedestrians in the image is fixed, the ratio of the pedestrian centroids’ sub-features in the feature map to the background’s sub-features is decreased, creating a denser pedestrian prediction frame in the feature map and increasing the demand on the prediction of the pedestrian detection frame’s confidence level. Additionally, because the resolution is increased by upsampling the high-resolution feature pyramid structure, the expanded resolution is a non-learnable upsampling mechanism. As a result, the neighboring sub-features have a high degree of similarity and produce redundant prediction frames that are larger than the prediction frame’s confidence threshold. In the original detection model, only one high-confidence prediction frame is produced for sparse targets in images. However, after the high-resolution feature pyramid structure, neighboring sub-features are similar to one another, and the number of prediction frames increases from one to multiple, all of which actually represent the same pedestrian. The succeeding NMS procedure requires more computation due to the significant increase in duplicate prediction frames, which also affects the detection model’s inference time.

In order to solve the aforementioned issues, we embed an attention mechanism module in the head of the detection model in this paper after the high-resolution feature pyramid structure. This causes the model to pay more attention to the most likely pedestrian subfeatures and less attention to the nearby sparse target subfeatures, allowing the detection model to filter out a large number of red targets.

**Figure 6:**
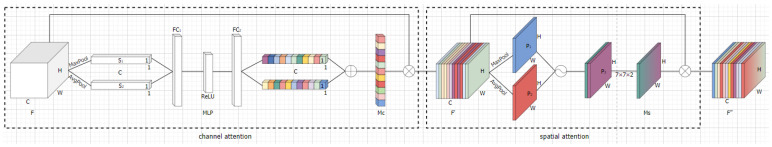
Flowchart for the CBAM attention mechanism module.

The literature [32] suggests an attention mechanism model (CBAM), combining channel attention and spatial attention, which enhances the detection model’s capacity to extract useful pedestrian feature information, cooperatively learns key local detail information in images, assigns higher weights to pedestrian features in the feature map and lower weights to the background, enhances the convolutional neural network’s attention to pedestrians, and enhances the feature extraction. Figure 6 depicts the entire flow diagram of the CBAM attention mechanism. The channel attention mechanism module processes the input image first to extract the feature weights of various feature channels in the feature map. The spatial attention mechanism module then processes the input image to obtain the spatial weighting information in the feature map to distinguish the significance of various spatial locations in the image. The CBAM module’s channel attention module, however, not only fails to take into account the mutual influence of neighboring feature channels but also adversely affects the prediction of channel attention due to the dimensionality reduction operation it uses.

**Figure 7:**
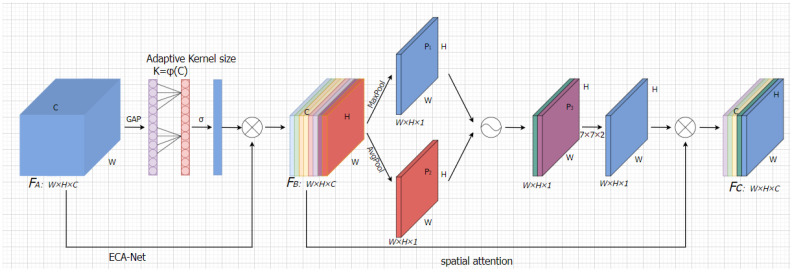
Flowchart for the ECSA-Net attention mechanism module.

In order to minimize the negative effects of downsampling and to successfully realize cross-channel interaction of images, an improved attention mechanism module called ECSANet (Efficient Channel and Spatial Attention Network) is built in this paper using CBAM as the baseline. Figure 7 depicts the ECSANet module’s network architecture. W×H is the width and height of $F_{A}$, and *C* represents the number of feature channels. The Efficient Channel Attention Network (ECANet) [33] module is used to extract the feature channel weight coefficients from the input picture before the input feature map, which has a dimension of  W×H×C, and is first compressed to 1×1×C via Global Average Pooling (GAP). A fast, one-dimensional convolution kernel of size K is then used to realize the local cross-channel interaction of the feature map and generate the channel attention weight coefficients after the cross-channel interaction. The image dimension is first compressed to 1×1×C by GAP. The channel attention weight coefficients are then produced after the local cross-channel interaction of the feature map using fast one-dimensional convolution with a convolution kernel of size K. The convolution kernel k is calculated adaptively by Equation (1), and we set γ and *b* to 2 and 1, respectively, during the experiments in this study. The range of cross-channel interactions is proportional to the channel dimension. Then, in order to produce image $F_{b}$, the feature channels of image $F_{b}$ are weighted using the feature channel weight coefficients. The spatial attention mechanism module receives $F_{b}$ as an input, and the spatial attention weights are generated using the spatial attention weights of the CBAM network. Global average pooling and global max pooling (GMP) are then applied to $F_{b}$, respectively, and the outputs are two feature maps of dimension W×H×1. The spatial attention weights are then obtained by a 7 × 7 ordinary convolution after connecting the two feature maps horizontally, and they are then weighted with the corresponding spatial positions of the feature map $F_{b}$. Afterward, the feature map $F_{c}$ is obtained after effective channel attention and spatial attention weighting.

(1) $$\left|k=\psi \left(C\right)={\left||\frac{log_{2}\left(c\right)}{\gamma }+\frac{b}{\gamma }|\right|}_{odd}\right|$$

## 4. Experimental Results and Analysis

The proposed approach is generated using Mobilenetv3’s pretrained weights on the CrowdHuman dataset. There are 300 training rounds in all, 50 of which are freeze training rounds with a 1 × 10^−2^ initial learning rate. Using the SGD optimizer, the batch_size is set to 16 for freeze training and to 8 for unfreeze training.

### 4.1. Experimental Environment

The environment configuration for this experiment was as follows: Intel(R) Core (TM) i7-10870H CPU @2.20HZ and NVIDIA GeForce RTX 2060 Ti GPU; the software environment for experiments and testing includes the Windows 10 operating system, CUDA 10.0 +cuDNN7.1 GPU gas pedal, and pytorch-based deep learning framework.

### 4.2. Experimental Evaluation Criteria

The two most commonly employed criteria for assessing target identification algorithms are precision rate and recall rate. The precision rate, abbreviated as $P_{r}$, is derived as stated in Equation (2). It is the percentage of targets accurately classified as belonging to the positive class among all targets judged to be in the category. The recall rate, abbreviated as $R_{e}$ and determined as given in Equation (3), is the percentage of targets correctly identified as belonging to the positive class among all positive class targets.

(2) $$P_{r}=\frac{T_{p}}{T_{p}+F_{p}}\times 100\%$$

(3) $$R_{e}=\frac{T_{p}}{T_{p}+F_{N}}\times 100\%$$

In the equation above, $T_{p}$ stands for the number of targets the algorithm correctly classified as belonging to the positive class, $F_{p}$ for the number of targets the algorithm wrongly classified as belonging to the positive class, and $F_{N}$ for the number of targets the algorithm incorrectly classified as belonging to the negative class.

Although precision and recall cannot be satisfied simultaneously in practical applications, the performance of the detection algorithm is assessed by combining precision and recall using the average precision $A_{p}$ value. Equation (4) displays the formula for calculating $A_{p}$:

(4) $$A_{p}=\sum_{1}^{N}P_{r}\left(k\right)\Delta R_{e}\left(k\right)$$

N is the total number of images in the test set, $P_{r}\left(k\right)$ is the $P_{r}$ value of image *k*, and triangle $R_{e}\left(k\right)$ denotes the transition from image *k*-1 to image *k* with $R_{e}$ value.

The performance of the detection network is typically assessed using the average accuracy $A_{p}$ value, or $m_{AP}$, which is generated as given in Equation (5), for specified jobs with Class *C* targets.

(5) $$m_{AP}=\frac{A_{p}}{C}$$

The precision rate and recall rate must be taken into account while using the detection algorithm in practice. The size of the *F*_1_ value can fully reflect the performance of the detection model, and the greater *F*_1_ value indicates the better detection performance of the model, as indicated in Equation (6). *F*_1_ is the sum of the precision rate and recall rate.

(6) $$F_{1}=2\times \frac{P_{r}\times R_{e}}{P_{r}+R_{e}}$$

In this study, the suggested algorithm is verified and examined using the aforementioned metrics.

### 4.3. Ablation Experiments

The ablation tests are carried out using the algorithm suggested in this research, using the YOLOv7 algorithm as the baseline. The tests compare and experiment with four different versions of the YOLOv7 algorithm: the original, YOLOv7+MobilenetV3, YOLOv7+HR-FPN, and YOLOv7+HR-FPN+ECSANet. The Adaptive NMS suppression algorithm is used to handle the redundant detection frames, and the threshold value of NMS is set to 0.5. When the IoU values of two detection boxes are less than 0.5, the unnecessary detection boxes are suppressed according to the ordinary NMS algorithm; otherwise, the NMS threshold value is adaptively adjusted according to In Table 1, the experimental findings are displayed.

**Figure 8:**
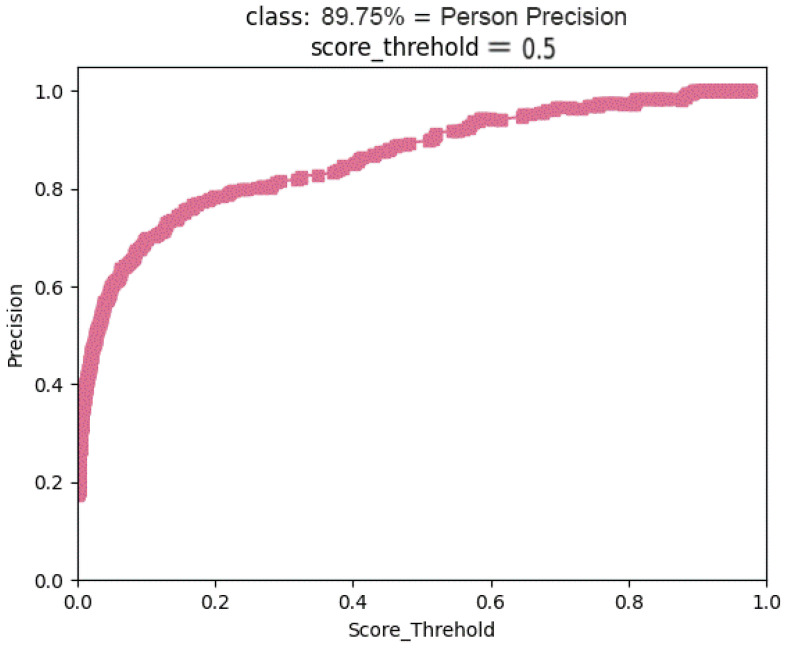
Accuracy curve of the algorithm proposed in this paper.

The experimental data in Table 1 demonstrate that the effectiveness of the detection model is significantly influenced by the backbone networks used. As compared to the original YOLOv7, the mAP of the YOLOv7+MobilenetV3 model is increased in this test by 2.61 percentage points, the model size is decreased to 1/6 of the original size, and the model inference speed is increased by 5 times. Additionally, it has been demonstrated through experimentation that the high-resolution feature pyramid module (HR) can significantly boost the detection efficiency of the detection algorithm model in dense scene detection of pedestrians. For pedestrians who are substantially concealed, the high-resolution map created by upsampling and fusing shallow feature maps can provide nearby and discernible deep features and increase pedestrian recall rates. The HR-FPN module significantly increases the detection rate of obscured pedestrians when compared to the baseline algorithm YOLOv7; however, the model inference speed drops to 26 FPS as a result of the increased feature map resolution, the increased number of redundant prediction frames, and the increased NMS computation, despite the HR-FPN module’s significant improvement in pedestrian detection rate. By adding this document to the detection network’s head. The average accuracy of the detection model is enhanced by embedding the suggested efficient attention module ECSANet in the head of the detection network, which also speeds up model inference by 12 frames per second. This is because the algorithm adapts its focus to emphasize pedestrian features more while suppressing unimportant information such as background noise thanks to the attention mechanism, which also lowers the computing cost of NMS. Although the running speed is slightly slower than the original YOLOv7 algorithm, the average accuracy has increased to 89.75%, as shown in Figure 8.

Additionally, to demonstrate the advancedness of the proposed detection algorithm for pedestrian detection in dense scenes, the proposed algorithm is compared with the most widely used pedestrian detection algorithms in order to confirm its efficacy. Table 2 displays the experimental results, with the bolded results in the table being the best outcomes overall.

The results above demonstrate that the improved YOLOv7 detection model is significantly more advanced in detecting obscured pedestrians in dense scenes, as shown by the data in Table 2, where the average detection accuracy of the proposed detection algorithm in this paper exceeds all other existing advanced algorithms in the table.

## 5. Discussion

**Figure 9:**
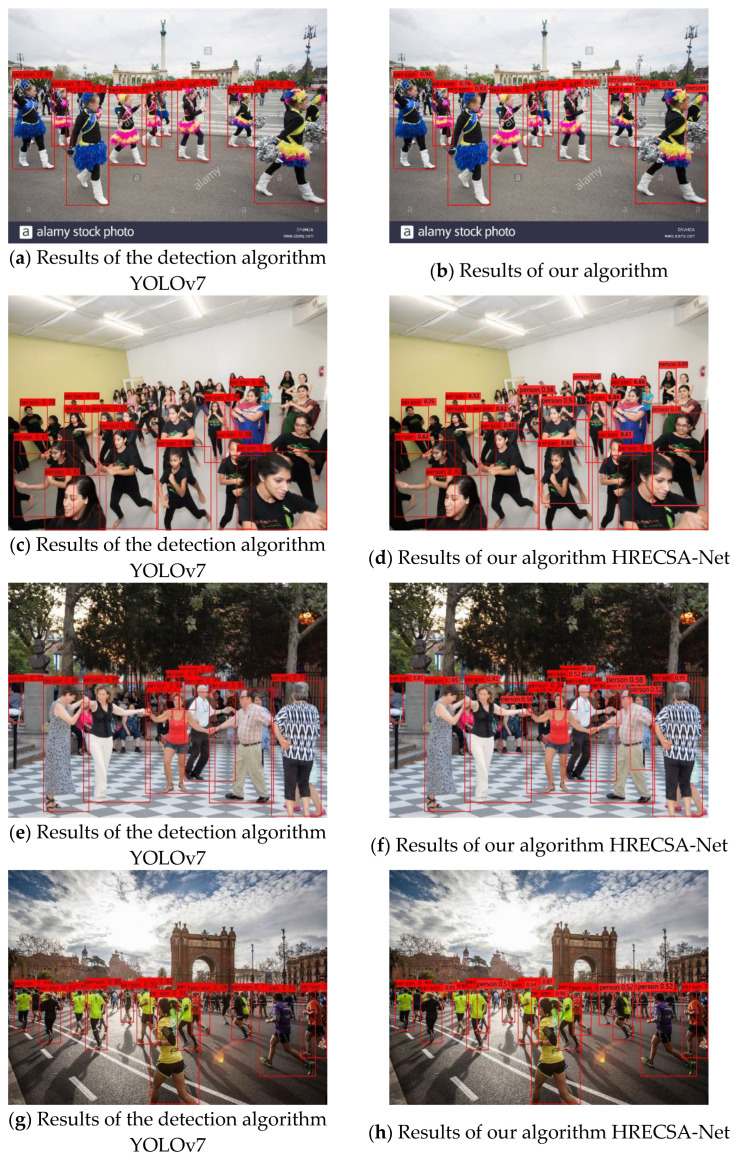
Comparison of the detection results for pedestrians. (**a**,**c**,**e**,**g**). Results of the detection algorithm YOLOv7; (**b**,**d**,**f**,**h**). results of our algorithm, HRECSA-Net.

YOLOv7 and the proposed approach are tested in the test set of the CrowdHuman pedestrian dense dataset, respectively, in order to more clearly demonstrate the advanced performance of this paper’s algorithm in recognizing veiled pedestrians in dense settings. The test results are displayed in Figure 9.

The YOLOv7 algorithm has more missed detection cases for obscured pedestrians and small-sized pedestrians in dense crowds, as shown above, where Figure 9a,c,e,g are the results of pedestrian detection using the YOLOv7 detection algorithm. Figure 9b,d,f,h on the right side are the output effect pictures after detection by the proposed algorithm in this paper. It is clear from this that the suggested algorithm is better able to handle the pedestrian recognition task in congested situations since the missed detection rate of small pedestrians and hidden pedestrians drops.

## 6. Conclusions

This paper proposes a lightweight pedestrian detection network called HRECSA-Net that is based on the improved YOLOv7 algorithm to address the common pedestrian occlusion problem in dense scenes. The algorithm is able to better handle the detection tasks of obscured pedestrians and small pedestrians by paying more attention to the valid information in the visible area. We use the lightweight convolutional neural network MobilenetV3 to replace the YOLOv7 algorithm model’s backbone network, which reduces the model size to 1/6 of the original size and significantly speeds up inference. Additionally, we create a high-resolution feature pyramid network to help the network extract data from the visible areas of obscured pedestrians and small-sized pedestrians more precisely and to lower the rate of missed detection of these pedestrians. Last but not least, this paper suggests an attention mechanism-based detection head for the redundant detection frames introduced by the high-resolution feature pyramid. It does this by embedding an improved efficient attention mechanism module, ECSA-Net, in the detection network head, which suppresses the extraction of unnecessary information and background noise, enabling the detection algorithm to filter out a significant number of redundant prediction frames and reducing the computation of the subsequent detection frames. The proposed method is capable of detecting people with severe occlusion, which considerably increases the algorithm’s detection accuracy for dense pedestrians, according to experimental results on the CrowdHuman dense pedestrian dataset. The proposed method, however, is unable to deal with the challenge of detecting pedestrians who are fully concealed for a brief period of time before reappearing.

## Figures and Tables

**Table 1:** Results of ablation experiments based on YOLOv7.

Algorithm	mAP	Speed (Frame · $s^{-1}$)
YOLOv7	80.25%	16
YOLOv7 + MobilenetV3	82.86%	190
YOLOv7 + MobilenetV3 + HR	85.27%	126
Ours (YOLOv7 + MobilenetV3 + HR + ECSA)	89.75%	138

**Table 2:** Comparison between the proposed algorithm and current advanced occluded pedestrian detection algorithms.

Algorithm	mAP	Speed (Frame · $s^{-1}$)
Faster R-CNN	71.5%	14
SSD	88.9%	20
Scaled-YOLOv4 [34]	85.7%	108
YOLOv5	83.1%	126
YOLOv7	80.25%	16
HRECSA-Net	89.75%	138
